# PARP16-Mediated Stabilization of Amyloid Precursor Protein mRNA Exacerbates Alzheimer’s Disease Pathogenesis

**DOI:** 10.14336/AD.2023.0119

**Published:** 2023-08-01

**Authors:** Jinghuan Wang, Qianwen Cheng, Yuyu Zhang, Chen Hong, Jiayao Liu, Xinhua Liu, Jun Chang

**Affiliations:** Pharmacophenomics Laboratory, Human Phenome Institute, Fudan University, Shanghai 201203, China

**Keywords:** PARP16, Alzheimer's disease, APP, mRNA stability, Endoplasmic reticulum

## Abstract

The accumulation and deposition of beta-amyloid (Aβ) are key neuropathological hallmarks of Alzheimer's disease (AD). PARP16, a Poly(ADP-ribose) polymerase, is a known tail-anchored endoplasmic reticulum (ER) transmembrane protein that transduces ER stress during pathological processes. Here, we found that PARP16 was significantly increased in the hippocampi and cortices of APPswe/PS1dE9 (APP/PS1) mice and hippocampal neuronal HT22 cells exposed to Aβ, suggesting a positive correlation between the progression of AD pathology and the overexpression of PARP16. To define the effect of PARP16 on AD progression, adeno-associated virus mediated-PARP16 knockdown was used in APP/PS1 mice to investigate the role of PARP16 in spatial memory, amyloid burden, and neuroinflammation. Knockdown of PARP16 partly attenuated impaired spatial memory, as indicated by the Morris water maze test, and decreased amyloid deposition, neuronal apoptosis, and the production of inflammatory cytokines in the brains of APP/PS1 mice. In vitro experiments demonstrated that the knockdown of PARP16 expression rescued neuronal damage and ER stress triggered by Aβ. Furthermore, we discovered that intracellular PARP16 acts as an RNA-binding protein that regulates the mRNA stability of amyloid precursor protein (APP) and protects targeted APP from degradation, thereby increasing APP levels and AD pathology. Our findings revealed an unanticipated role of PARP16 in the pathogenesis of AD, and at least in part, its association with increased APP mRNA stability.

## INTRODUCTION

Alzheimer’s disease (AD) is a complex neurodegenerative disorder clinically characterized by progressive memory and cognitive deficits [1, 2]. Accumulation of beta-amyloid (Aβ) plaques and phosphorylated tau-containing neurofibrillary tangles are two major neuropathological hallmarks of AD [3-5]. Furthermore, Aβ is produced by β- and γ-secretase-mediated cleavage of amyloid precursor protein (APP) [6]. In AD, excessive Aβ production and decreased catabolism lead to Aβ aggregation and trigger a series of pathological changes, including chronic inflammatory reaction, cell death, and progressive synaptic loss [7] [8]. Despite great efforts and investment, since the pathogenesis of AD is complex, effective early prevention and treatment remain elusive [9-11].

Multiple studies have indicated that endoplasmic reticulum (ER) stress occurs in AD brains [12, 13]. During AD, the continuous accumulation of Aβ or p-tau results in a drastic alteration in ER calcium homeostasis, abnormal protein folding, and ER stress, provoking ER stress-dependent cell death and synaptic depression [14]. Under mild ER stress, the unfolded protein response (UPR) is a cellular response to ER stress and has a pro-adaptive role. However, in prolonged or aggravated ER stress, the UPR-pro-apoptotic branch and pro-inflammatory mediators are triggered, which is considered a possible cause of neurodegeneration [15, 16]. Meanwhile, ER stress promotes Aβ neurotoxicity and enhances tau protein phosphorylation, which further triggers UPR in neurons, generating a vicious cycle that drives the progression of AD. The role of the UPR in the neuropathology of AD has been established; therefore, inhibition of specific ER mediators may contribute to the treatment and prevention of AD.

PARP16 (also known as ARTD15), a Poly(ADP-ribose) polymerase (PARP), is the only known tail-anchored ER transmembrane protein with a cytosolic catalytic domain. PARP16 transduces ER stress through two components of the UPR: protein kinase RNA (PKR)-like ER kinase (PERK) and inositol-requiring protein-1 (IRE1). Growing evidence indicates that PARP family members have long been known to regulate RNA binding and mRNA processing; for instance, PARP1 can regulate inflammatory gene expression by increasing mRNA stability [17], and the cytoplasmic PARP family member PARP14 is also known to regulate mRNA stability [18]. These examples highlight the importance of PARP family members in mRNA processing and protein abundance, showing that the dysregulation of PARP activity in these processes can affect disease states [19]. Our previous studies have confirmed that PARP16 plays an important role in homeostasis in vascular diseases [20, 21], whether PARP16 is involved in the pathological processes of AD has not been investigated.

Here, we address the possible contribution of PARP16 in the context of AD. We found that PARP16 was increased in both Aβ-treated cultured neurons in vitro and in the hippocampi of APP/PS1 mice. PARP16 deficiency in the nervous system reduced amyloid load and improved neuronal damage and memory deficits in a mouse model of AD. Meanwhile, PARP16 deficiency rescued Aβ-induced ER stress and pro-survival signals in neurons. Our results uncovered a previously unanticipated role of PARP16 in AD and suggested that strategies to inhibit its activity may be useful in halting disease progression.

## MATERIALS AND METHODS

### Antibodies, chemicals, and reagents

The following antibodies were used: PARP16 (ARP33751) was obtained from Aviva (Aviva Systems Biology, USA); Aβ 6E10 (800304) was obtained from BioLegend (BioLegend, Inc. USA); sAPPβ (10321) and sAPPα (11088) were obtained from IBL (Immuno-Biological Laboratories Co., Ltd., Japan); p-IRE1α (PA1-16927) was obtained from ThermoFisher (Thermo Fisher Scientific Inc., USA); XBP-1 (ab37152), and Bcl-2 (ab196495) were obtained Abcam (Abcam); APP (2450), p-PERK (3179), p-eIF2α (3597), Bax (2772), Caspase 3 (9662), and p53 (2524) were purchased from CST (Cell Signaling Technology, Inc., USA); COX-2 (A3560), IL-1β (A1112) and IL-6 (A0286) were obtained from ABclonal (ABclonal Technology Co.,Ltd. China); iNOS (18985-1-AP), BIP (11587-1-AP), Calnexin (10427-2-AP), β-tubulin (10094-1-AP), β-Actin (66009-1-Ig), and GAPDH (60004-1-Ig) were purchased from Proteintech (Proteintech Group, Inc., USA). Actinomycin D was purchased from Linkchem (Shanghai LinkChem Technology Co., Ltd. China). MG132 was purchased from Meilunbio (Dalian, China). 3-Methyladenine (3-MA) and tauroursodeoxycholic acid (TUDCA) were obtained from MCE (MedChemExpress, USA). Aβ_1-42_ (A9810) was purchased from Sigma-Aldrich (Sigma-Aldrich, USA).

### Animal studies and virus injection

The animal study protocol was approved by the Institutional Ethics Committee of Fudan University, China. Adeno-associated virus (AAV)-shRNA *Parp16* (AAV, 1.5 × 10^12^ genomic copies per mL), and AAV-scramble shRNA Control (AAV-scRNA) (AAV, 1.5 × 10^12^ genomic copies per mL) were obtained from HanBio (Shanghai, China). Four-month-old male C57BL/6J (WT) and APP/PS1 mice were randomly divided into three groups: WT group (WT + AAV-scRNA), APP/PS1 group (APP/PS1 + AAV-scRNA), and shRNA *Parp16*-treated group (APP/PS1 + AAV-shRNA *Parp16*). First, 3 μL AAV-scRNA or AAV-shRNA *Parp16* were injected into the lateral ventricle via stereotaxis technology. The coordinates were as follows [22]: anteroposterior, -2.3 mm; mediolateral, ±1.8 mm; dorsoventral, -2.0 mm. The needle was kept in the injection site for another 5 min and then withdrawn slowly to prevent solution leakage. Mice were allowed to recover on a heating pad and were returned to the animal facility. The WT and APP/PS1 groups received equal volumes of AAV-scRNA. At 4.5 months post-injection, the mice were tested in the Morris water maze (MWM).

### Morris Water Maze

The MWM test was conducted for six consecutive days according to a previously described protocol, with minor modifications [22]. Briefly, the MWM test in our experiment included the following two components: a 5-day consecutive place navigation test and a 1-day spatial probe test. The MWM task was resumed on the final day (day 6), and escape latency was recorded. After 5 days of continuous training, the platform was moved, and the mice were gently released into the quadrant opposite to that which had previously contained the platform. The mice were allowed to swim for 60 s, and their duration in the target quadrant (quadrant containing the platform) and number of platform crosses were recorded. The escape latencies determined during the place navigation test and the spatial probe test were used to assess visual-spatial and learning abilities, respectively. The spatial memory ability of the mice was assessed based on the time spent in the target quadrant and number of platform crossings during the spatial probe test (on day 6).

### Measurement of cerebral blood flow (CBF)

CBF of mice from various groups was determined using the PeriCam PSI System (Primed, Sweden). Briefly, mice were anesthetized with 1% pentobarbital sodium (Sigma-Aldrich, USA) and placed on a stereotaxic apparatus. A cross-skin incision was made on the head to expose the entire skull. PeriCam PSI System scanning (2.0 × 1.4 cm) was performed on the intact skull for approximately 1 min. The mean blood perfusion of the brain was analyzed using Pimsoft software (Pimsoft).

### Histological staining

Coronal sections were rinsed with distilled water and stained with alum hematoxylin, followed by differentiation with 0.3% acid alcohol. After rinsing with distilled water, the sections were stained with eosin for 2 min and dehydrated for mounting. TUNEL assays (ThermoFisher Scientific) and Nissl staining (Servicebio, China) were performed according to the manufacturer’s instructions.

Immunostaining was performed using rabbit monoclonal anti-PARP16 (ARP33751, Aviva Systems Biology, USA) and anti-6E10 (800304, BioLegend). Normal rabbit IgG (2729, Cell Signaling Technology) was used as negative control. Secondary antibodies conjugated with horseradish peroxidase (ab64264, Abcam) were used and visualized according to standard protocols.

### Immunofluorescence

For immunofluorescence, tissue sections or cell slides were incubated with primary rabbit anti-Bcl-2 (ab196495, Abcam), anti-IL-1β (A1112, ABclonal), and mouse anti-APP antibodies (2450, Cell Signaling Technology), followed by incubation with Goat anti-Mouse IgG Cross-Adsorbed Secondary Antibody labeled with Alexa Fluor 488 and Goat anti-Rabbit IgG Cross-Adsorbed Secondary Antibody labeled with Alexa Fluor 568 (ThermoFisher Scientific). The sections or slides were imaged using a fluorescence microscope (Zeiss).

### ELISA

Homogenized cortical and hippocampal brain samples were collected. The level of Aβ in the tissue was assayed using a Mouse Aβ_1-42_ ELISA kit (KMB3441, ThermoFisher Scientific) according to the manufacturer’s instructions. The levels of tumor necrosis factor (TNF-α) and interleukins (IL)-1β and IL-6 in the tissue were assayed using an ELISA kit (DAKEWE, China) according to the manufacturer’s instructions. The results are presented as ng/mg total tissue protein.

### Cell culture

Mouse hippocampal neuronal cell lines HT22 and HEK293 cells stably transfected with APP cDNA harboring the Swedish mutation KM670/671NL (HEK/APPsw) (HEK-APP) were cultured at 37°C in DMEM-F12 medium or Dulbecco’s modified Eagle’s medium (DMEM) supplemented with 10% fetal bovine serum (FBS) and an antibiotic solution (100 U/ml penicillin, 0.1 mg/ml streptomycin) in a humidified atmosphere in the presence of 5% CO_2_.

### Small interfering RNA transfection in vitro

Mouse Parp16 (5′-CCUCCAAGAUCCUGACAAUTT-3′), human PARP16 (5′- CCUCAAAGGUCCUGACA AUTT-3′), and negative control small interfering RNA (siRNA) were obtained from GenePharma (Shanghai, China). Cells at approximately 70% confluence were added to the prepared Lipofectamine RNA iMAX (ThermoFisher Scientific) and siRNA complexes. After incubating for 12 h, the medium was removed. Three days later, PARP16 knockdown efficiency was evaluated.

### Western Blot

The hippocampi and cortices were dissected and immediately frozen in liquid nitrogen. The tissue and cell samples were extracted with radioimmunoprecipitation assay (RIPA) buffer, supplemented with 1% protease inhibitor cocktail and 1% phosphatase inhibitor cocktail (APExBIO, USA), and centrifuged at 12,000 rpm at 4°C for 20 min. The supernatant was collected, and the total protein concentration in each sample was determined using a bicinchoninic acid (BCA) assay (ThermoFisher) according to the manufacturer’s instructions. Equal amounts of protein samples were separated by sodium dodecyl sulfate-polyacrylamide gel electrophoresis and transferred to nitrocellulose membranes (Millipore). The membranes were blocked with 5% skimmed milk (w/v) in Tris-buffered saline supplemented with 0.1% Tween 20 (TBST) for 1 h at room temperature and then incubated with the corresponding primary antibodies at 4°C overnight. The membranes were then washed and incubated for 1 h at room temperature with the appropriate horseradish peroxidase (HRP)-labeled secondary antibody (Jackson ImmunoResearch Inc., USA). Protein-specific signals were detected using a Bio-Rad Imager (Bio-Rad, Hercules, CA, USA), and the bands were quantified by densitometric analysis using ImageJ software (NIH).

### Real-time quantitative polymerase chain reaction (RT-qPCR)

Total RNA from cells and tissues was isolated using TRIzol, reverse transcribed, and subjected to real-time PCR. Primer sequences of the genes were as follows: Mouse Parp16: 5’-CTCCTTGGCCAGACCCTTAG-3’ and 3’-GATAGAGGGACATCGCGACC-5’; Mouse App:5’-CTCCGTGTGATCTACGAGCG-3’ and 3’-GGTCTTGGTTTCCGTCAGCG-5’; human APP:5’-GT GGCATTCTTTTGGGGCTG-3’ and 3’-GCCCACCAT GAGTCCAATGA-5’. The level of expression was quantified using the 2^-∆∆Ct^ method with GAPDH as a control for normalization.

### RNA stability determined by the 5-ethynyl Uridine (EU) labeling assay

The 5-ethynyl Uridine (EU) labeling assay was performed using the Click-iT Nascent RNA Capture Kit according to the manufacturer’s instructions [23]. Briefly, cells were first treated with EU (0.1 mM) for 24 h; then, EU was removed, and the cells were cultured for another 24 h. Both samples were harvested before or after EU removal to extract RNAs. Then, EU-labeled RNAs were biotinylated by click reaction and isolated using streptavidin beads (ThermoFisher Scientific). The stability of the mRNAs was determined as the percentage of labeled mRNA after removal of EU at different time points divided by the percentage of labeled mRNA before removal.

### Nuclear run-on

Nuclear runs were performed as previously described [24]. First, the cell nuclei were extracted and used for the run-on reaction. Subsequently, the nuclei were added with the same volume of transcription buffer (20 mM Tris-HCl, 200 mM KCl, 5 mM MgCl_2_, 4 mM dithiothreitol (DTT), 4 mM each of ATP, CTP and GTP, 1 mM biotin-16-UTP, 200 mM sucrose, and 20% glycerol), and incubated for 30 min at 30°C, then the de novo synthesized transcription was purified by DynabeadsTM M-280 (ThermoFisher Scientific). Purified RNAs were subjected to reverse transcription and quantification.

### RNA immunoprecipitation and RNA pull down

RNA immunoprecipitation (RIP) was performed using an RNA Immunoprecipitation Kit (GENESEED, China) according to the manufacturer’s instructions. Crosslinked RIP was performed as previously described with minor modifications. Briefly, HEK-APP cells were lysed, and the supernatant was mixed with antibody PARP16 and protein A/G magnetic beads, followed by overnight incubation at 4°C. The beads were then washed with a buffer to remove non-specific binding proteins. The RNAs in the beads were eluted and detected using RT-PCR.

An RNA pull-down assay was performed to detect the binding of the RNA-binding proteins from whole Aβ_1-42_ treated-HT22 cells extracts. In short, in vitro biotin-labeled APP or mutated-APP mRNA (GenePharma, China) were mixed with lysates from Aβ_1-42_ treated-HT22 cells, and the RNAs-protein complex was targeted with streptavidin beads (ThermoFisher Scientific). The beads were then washed with buffer, and western blot analysis was performed on the co-precipitated proteins.

### Statistical analysis

Data are expressed as the mean ± S.D. Differences in means were analyzed for normality using one-way ANOVA with the Tukey or Bonferroni post hoc tests for multiple groups, and Student’s t-test was used when comparing two groups. Non-parametric comparisons (Mann-Whitney U Test or Kruskal-Wallis Test) were used for non-normally distributed data or n < 6. All analyses were performed using the GraphPad Prism 8.0 statistical software package, and a *p* value < 0.05 was considered statistically significant.

## RESULTS

### APP/PS1 mice show increased PARP16 expression, and ablation of PARP16 ameliorates cognitive decline and AD pathology in the APP/PS1 mouse model

**Figure 1.**
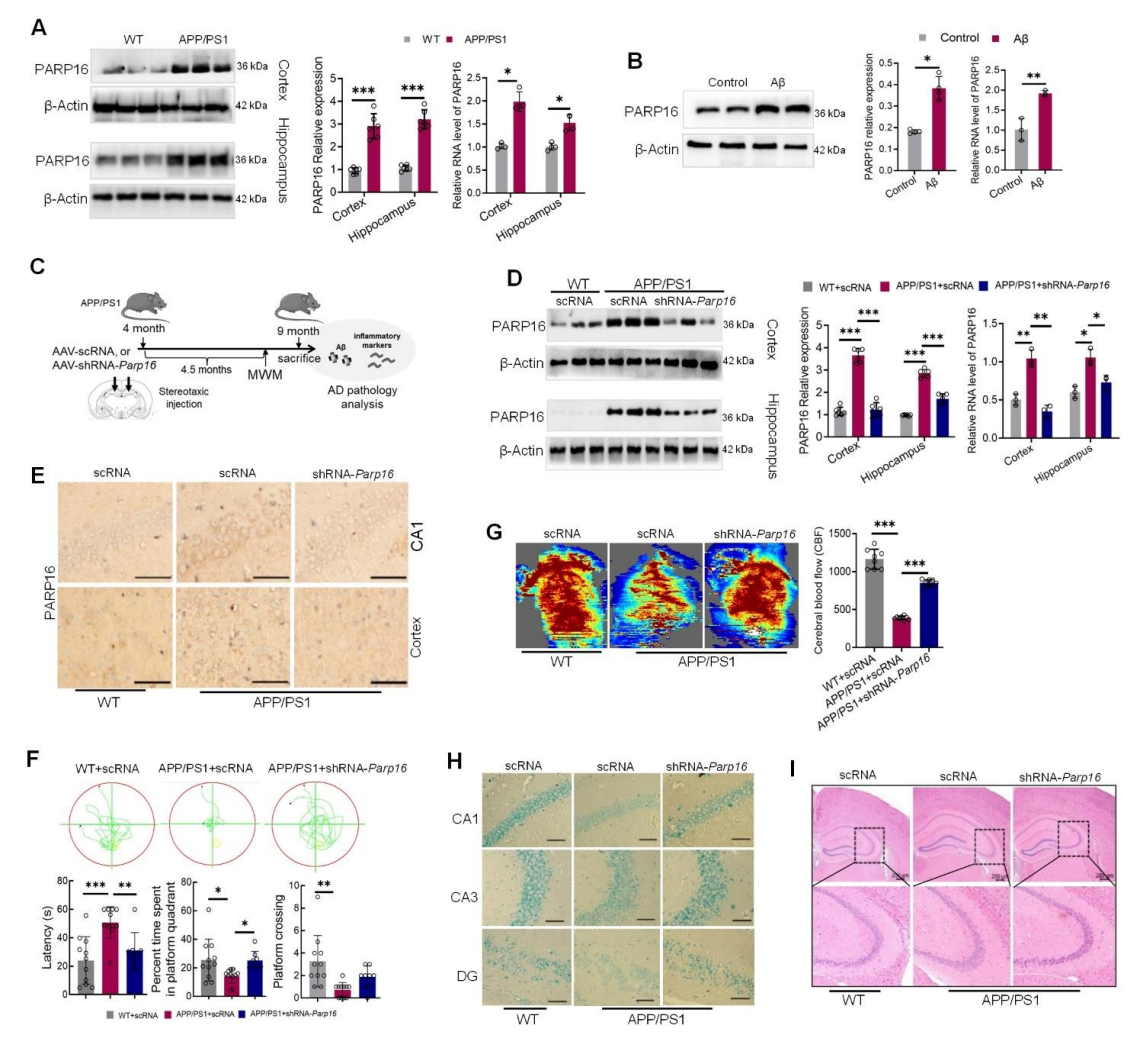
**Knockdown of PARP16 improves behavioral performance in experimental models of APP/PS1 mice**. (**A**) Amyloid precursor protein (APP)/PS1 mice enhanced PARP16 protein (n=6) and mRNA levels (n=4) in the hippocampi and cortices. Data are presented as the mean ± S.D. ^*^p < 0.05, ^***^p < 0.001. (**B**) Aβ_1-42_ enhanced PARP16 protein and mRNA levels in HT22 cells. Data are presented as the mean ± S.D, ^*^p < 0.05, ^**^p < 0.01. At least three experiments were repeated. (**C**) Experimental design of knockdown of PARP16 prevention study in APP/PS1 mice. (**D**) APP/PS1 and WT mice were injected intracerebroventricularly (i.c.v.) with an AAV-expressing shRNA targeting PARP16 (shRNA-*Parp16*) or AAV-scramble control shRNA (scRNA, used as a control). PARP16 expression was determined by western blots (n=6) and qPCR (n=3) in the hippocampi and cortices of WT and APP/PS1 mice. Data are presented as the mean ± S.D. ^*^p < 0.05, ^**^p < 0.01, ^***^p < 0.001. (**E**) Representative photomicrographs of immunohistochemistry staining for PARP16 in the hippocampi and cortices of WT and APP/PS1 mice. (**F**) Escape latency, percentage of time spent in the target quadrant, and the platform-crossing frequency in the Morris water maze (MWM) probe phase were detected. Data are presented as the mean ± S.D. ^*^p < 0.05, ^**^p < 0.01, ^***^p < 0.001, n = 8-11/group. (**G**) Typical cerebral blood flow (CBF) maps and quantification of CBF. Data represent the mean ± S.D. ^***^p < 0.001, n = 8. (**H**) Representative images of Nissl staining in the CA1, CA3, and DG regions of the hippocampus in WT and APP/PS1 mice. (**I**) H&E staining was performed to assess pathological changes in the cortex of APP/PS1 mice.

We first examined the state of PARP16 in different brain regions from 9-month-old APP/PS1 mice and compared the measurements with those of age-gender matched WT mice. As shown in Fig. 1A, APP/PS1 mice showed a significant increase in the mRNA and protein levels of PARP16 in both the hippocampus and cortex, while the level of PARP16 was increased in immortalized mouse hippocampal HT22 neurons exposed to oligomeric Aβ_1-42_ peptide (Fig. 1B). At the age of 8 months, APP/PS1 transgenic mice develop abundant plaques in the hippocampus and cortex and show deficits in learning and memory [25, 26]. Based on our observations regarding PARP16 in AD models, we determined the prophylactic effect of PARP16 ablation on cognitive deficits and AD neuropathology in APP/PS1 mice. The animal experimental design is depicted in Fig. 1C; we intraventricularly injected AAV-shRNA-Parp16 or AAV-scRNA to 4-month-old male APP/PS1 mice. As controls, age-matched male WT mice were intraventricularly injected with AAV scRNA. As shown in Fig. 1D, at 5 months post-injection, AAV-shRNA-*Parp16* administered-APP/PS1 mice showed a significant decrease in the mRNA and protein levels of PARP16 in both the cortex and hippocampus. Immunostaining further demonstrated a decrease in PARP16 positive cells (Fig. 1E).

We investigated whether AD-associated impairments in memory could be alleviated by reducing PARP16 levels and performed the MWM test. The mice underwent 5 days of hidden platform training (four sessions per day) after visible platform training, and there were no statistical differences in latencies to find the platform between the AAV-shRNA-*Parp16* and AAV-scRNA groups. After the completion of training, a probe trial was performed in which the platform was removed, and indices of spatial memory were measured. Representative movement tracks of the mice on day 6 in the place navigation component of the MWM are shown in Fig. 1F. Knockdown of PARP16 in mice markedly shortened the escape latency and increased the percentage of time spent in the target quadrant compared to APP/PS1 mice. However, there were no statistical differences in the incidence of crossing the removed-platform area between the AAV-shRNA-*Parp16* and AAV-scRNA APP/PS1 groups (Fig. 1F). These results suggest that knockdown of PARP16 in APP/PS1 mice partly alleviates spatial memory deficits.

Several studies have shown that reduction in cerebral blood flow (CBF) plays an important role in disease progression [27, 28]. We observed a reduction in regional CBF in APP/PS1 mice. Surprisingly, CBF reduction was elevated by PARP16 knockdown (Fig. 1G). The Nissl staining results suggested that the hippocampal neurons in APP/PS1 mice exhibited a loss of Nissl bodies, swollen neurons, and a disordered cell arrangement compared to the WT group; however, in AAV-shRNA-*Parp16*-administered APP/PS1 mice, the Nissl bodies were relatively abundant and clear in color (Fig. 1H). According to the results from H&E staining, the APP/PS1 group displayed lower neuronal cell density and disordered cell arrangements in the hippocampus compared with the WT group. After knockdown of PARP16, the neuronal density was increased in the CA3 regions (Fig. 1I). These results indicated that the knockdown of PARP16 improved the morphology of APP/PS1 mice.

**Figure 2.**
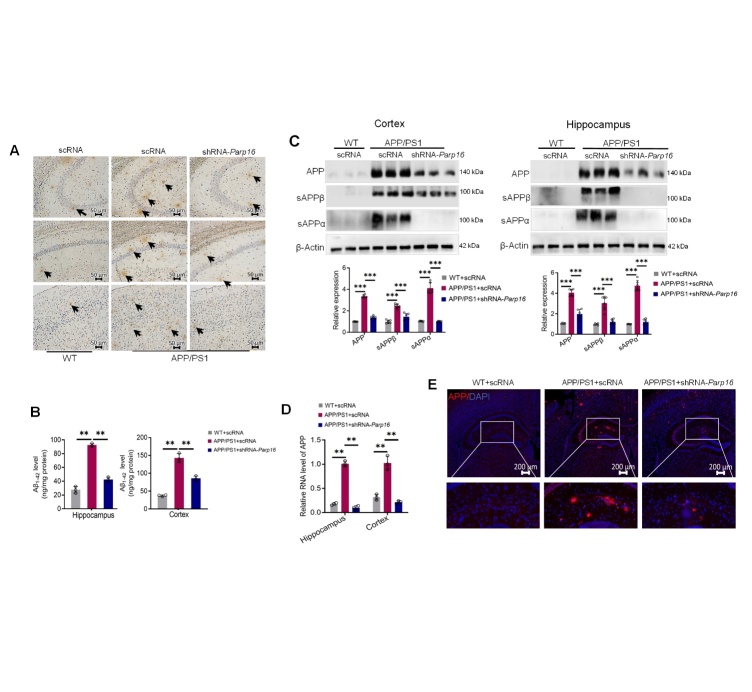
**Knockdown of PARP16 decreases amyloid precursor protein (APP) levels and Aβ burden in APP/PS1 mice**. (**A**) APP/PS1 and WT mice were injected intracerebroventricularly (i.c.v.) with an AAV-expressing shRNA targeting PARP16 (shRNA-*Parp16*) or AAV-scramble control shRNA (scRNA, used as a control). Representative images of Aβ plaques were visualized using 6E10 in WT and APP/PS1 mice. Scale bar, 50 μm. (**B**) Total concentration of soluble Aβ_1-42_ in the hippocampi and cortices from WT and APP/PS1 mice were measured by using ELISA. Data are presented as mean ± S.D. ^**^p < 0.01, n = 3. (**C**) Western blots and quantitative analyses of APP, sAPPα, and sAPPβ in the hippocampi and cortices of mice. Data are presented as mean ± S.D. ^***^p < 0.001, n = 6. (**D**) qPCR quantitative analyses of APP mRNA in the hippocampi and cortices. Data are presented as mean ± S.D. ^**^p < 0.01, n = 3. (**E**) Representative photomicrographs of immunofluorescence staining for APP in the cortex of WT and APP/PS1 mice. Scale bar, 200 μm.

### Ablation of PARP16 reduces Aβ plaques and oligomers in the cortex and hippocampus of APP/PS1 mice

Overloading of Aβ plaques is the major pathology in the brains of APP/PS1 mice [29, 30]. The formation of these plaques in the cortical and hippocampal regions is directly related to learning and memory deficits in AD [31]. Aβ plaques were assessed by immunohistochemistry in shRNA-*Parp16*-administered APP/PS1 mice and control APP/PS1 mice. Amyloid plaque loads in the cortex and hippocampus were significantly increased in APP/PS1 mice at 9 months of age. Ablation of PARP16 reduced the accumulation of Aβ plaques compared with that in APP/PS1 mice (Fig. 2A). To detect the relative levels of Aβ, we performed Aβ_1-42_ quantification in hippocampal and cortical homogenates using a specific Aβ_1-42_ ELISA assay. Consistently, ELISA showed lower levels of Aβ_1-42_ in the hippocampi and cortices of shRNA-*Parp16*-administered APP/PS1 mice than in control APP/PS1 mice (Fig. 2B). To further confirm these results, tissue homogenates from the hippocampi and cortices were used to detect the expression of APP, sAPPα, and sAPPβ by western blotting, and it was found that PARP16 knock-down presented lower levels of APP, sAPPα, and sAPPβ in both hippocampi and cortices (Fig. 2C). Consistently, the APP mRNA levels also decreased in the hippocampi and cortices of APP/PS1-shRNA-*Parp16* mice (Fig. 2D). Furthermore, we used immunofluorescence staining to verify our results; APP immunodensity increased in the APP/PS1 mouse brain. In contrast, the intensity of APP staining was significantly decreased in APP/PS1-shRNA-*Parp16* mice (Fig. 2E). Taken together, these results indicate that PARP16 deficiency significantly affects plaque formation in various brain regions.

**Figure 3.**
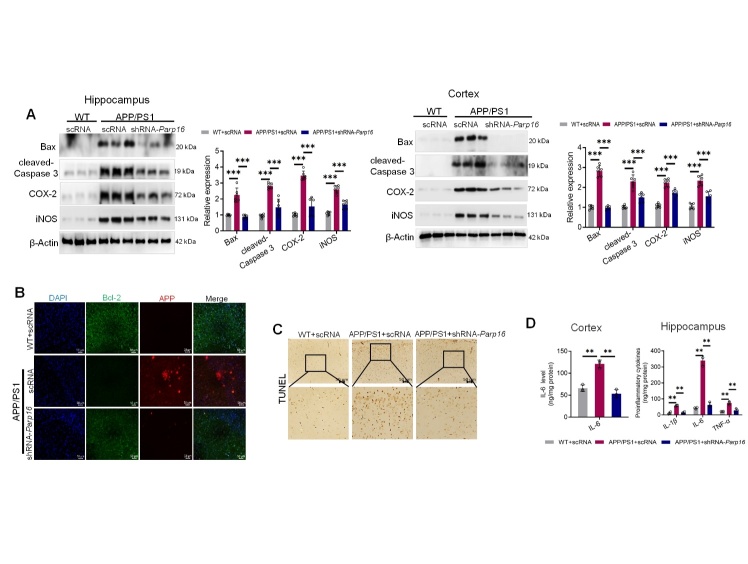
**The knockdown of PARP16 alleviates cell apoptosis and neuroinflammation**. (**A**) APP/PS1 and WT mice were injected intracerebroventricularly (i.c.v.) with an AAV-expressing shRNA targeting PARP16 (shRNA-*Parp16*) or AAV-scramble control shRNA (scRNA, used as a control). Western blots and quantitative analyses of apoptosis (Bax and cleaved-Caspase 3) and pro-inflammatory factors (COX-2 and iNOS) in the hippocampi and cortices of WT and APP/PS1 mice are also shown. Data represent the mean ± S.D. ^***^p < 0.001, n = 6. (**B**) Representative photomicrographs of immunofluorescence staining for Bcl-2 and APP. (**C**) Neuronal apoptosis was detected using a TUNEL staining assay in the cerebral cortex of WT mice and APP/PS1 mice. (**D**) TNF-α, IL-6, and IL-1β in the hippocampi, and IL-6 in the cortices were evaluated by using ELISA. Data represent the mean ± S.D. ^**^p < 0.01, n = 3.

**Figure 4.**
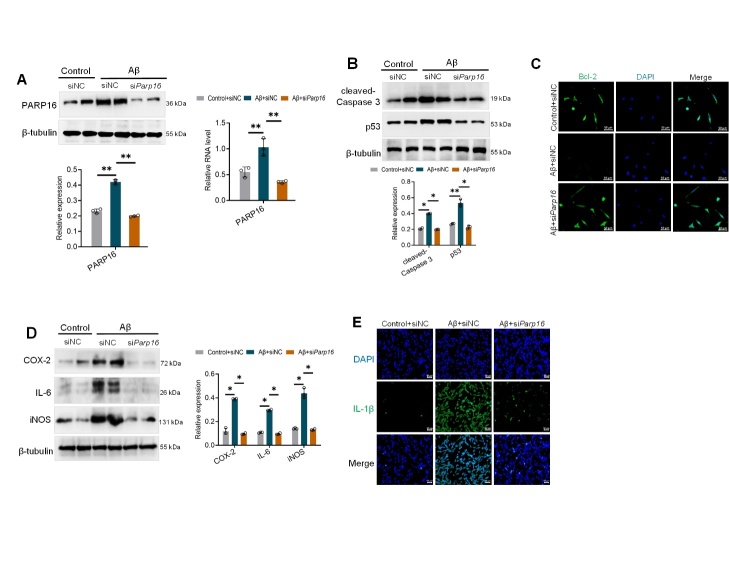
**Knockdown of PARP16 alleviates Aβ_1-42_-induced HT22 cell injury**. (**A**) HT22 cells transfected with negative control (siNC) mimics or siRNA *Parp16* were treated with or without Aβ_1-42_, and PARP16 expression was measured by RT-qPCR and western blotting. Data represent the mean ± S.D. ^**^p < 0.01. At least three experiments were repeated. (**B**) Western blotting was performed to assess the protein levels of apoptosis factors p53 and cleaved-Caspase 3. Data represent the mean ± S.D. ^*^p < 0.05, ^**^p < 0.01. At least three experiments were repeated. (**C**) Representative photomicrographs of immunofluorescence staining for Bcl-2. (**D**) Western blotting was performed to assess the protein levels of inflammation mediators COX-2, iNOS, and IL-6. Data represent the mean ± S.D. ^*^p < 0.05. At least three experiments were repeated. (**E**) Representative photomicrographs of immunofluorescence staining for IL-1β.

### Ablation of PARP16 reduces cell death and neuroinflammation in APP/PS1 mice

Cell death and neuroinflammation have been shown to contribute to neurodegeneration in AD [32]. To further address whether ablation of PARP16 reduced cell death and inflammatory stress, apoptosis and inflammation markers in the hippocampi and cortices of APP/PS1 mice were determined using western blotting. We showed that APP/PS1 overtly upregulated the levels of the pro-apoptotic markers Bax, cleaved-Caspase 3, and the pro-inflammatory markers COX-2 and iNOS (Fig. 3A), while suppressing the level of anti-apoptotic marker Bcl-2, as shown by immunofluorescence staining (Fig. 3B). These effects were abrogated by the knockdown of PARP16. Terminal deoxynucleotidyl transferase dUTP nick-end labeling (TUNEL) staining indicated that the APP/PS1 mutation overtly promoted apoptosis, and the effect was negated by knockdown of PARP16 (Fig. 3C). ELISA analysis showed lower levels of TNF-α, IL-1β, and IL-6 in the hippocampi, and lower level of IL-6 in the cortices of shRNA-*Parp16*-administered APP/PS1 mice than in control APP/PS1 mice (Fig. 3D). Therefore, these results indicate that ablation of PARP16 reduces cell death and neuroinflammation in APP/PS1 mice.

### Ablation of PARP16 protects neurons from damage induced by Aβ (1-42) in vitro

Extracellular Aβ is internalized into neural cells, and this internalization is associated with cell death in the brain [33]. We prepared Aβ (1-42) aggregates by incubating Aβ (1-42) monomers at 37°C overnight and then diluted the peptide stock with cell culture medium. To determine whether knockdown of PARP16 affects neural cell injury, we used siRNA *Parp16* to knock down PARP16 in Aβ_1-42_-treated HT22 cells (Fig. 4A). To explore the cell death signaling pathway by which PARP16 knockdown blocked Aβ-induced neural cell death, the expression levels of apoptotic molecules were measured in HT22 cells. Remarkably, knockdown of PARP16 significantly recovered HT22 cells from the apoptotic state in terms of cleaved-Caspase 3, p53, and Bcl-2 expression (Fig. 4B and 4C). Furthermore, we observed a significant increase in the levels of many inflammatory factors in HT22 cells exposed to Aβ, including iNOS, COX-2, IL-6, and IL-1β, which was reduced by knockdown of PARP16 (Fig. 4D and 4E). Taken together, these findings suggest that ablation of PARP16 blocks Aβ-induced neural cell death and inflammation.

**Figure 5.**
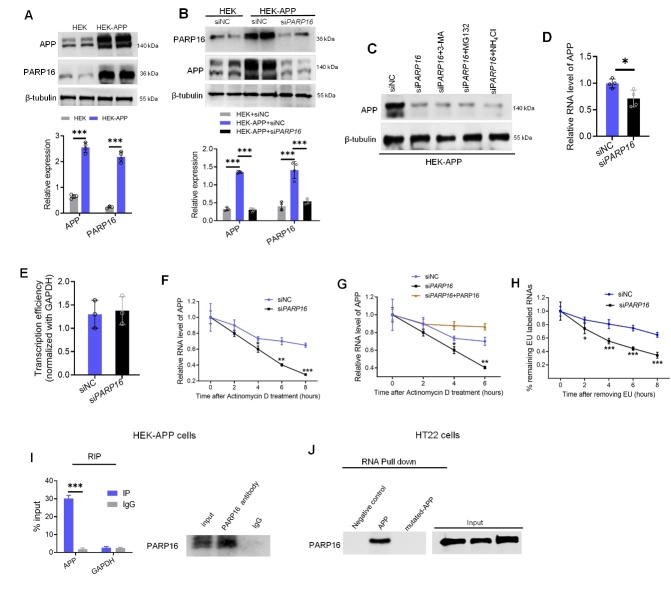
**PARP16 binds and stabilizes APP mRNA**. (**A**) PARP16 expression was increased in HEK-APP cells. Data are presented as the mean ± SD, ^***^p < 0.001. At least three experiments were repeated. (**B**) Knockdown of PARP16 attenuated APP expression. HEK and HEK-APP cells were transfected with negative control (siNC) mimics or siRNA *PARP16*, and PARP16 and APP were measured by western blotting. Data represent the mean ± S.D, ^***^p < 0.001. At least three experiments were repeated. (**C**) PARP16 knocked down-HEK-APP cells were treated with MG132, 3-MA, or NH_4_Cl, and APP levels were measured by western blotting. (**D**) Knockdown of PARP16 attenuated APP mRNA levels. HEK-APP cells were transfected with negative control (siNC) mimics or si*PARP16*, and APP mRNA was measured by qPCR. Data are presented as mean ± S.D, ^*^p < 0.05, n = 4. (**E**) The nuclear run-on assay was used to determine PARP16 knockdown on APP transcription efficiency. Data are presented as the mean ± S.D, n = 3. (**F**) Control HEK-APP cells, and PARP16 knocked down-HEK-APP cells were treated with the transcription inhibitor Actinomycin D (5 μg/ml). APP mRNA levels were quantified using RT-qPCR. Data are presented as the mean ± S.D, ^*^p < 0.05, ^**^p < 0.01, ^***^p < 0.001, n = 3. (**G**) Control HEK-APP cells, PARP16 knockdown cells, and knockdown HEK-APP cells with PARP16 re-expressed were treated with the transcription inhibitor Actinomycin D (5 μg/ml). APP mRNA levels were quantified using RT-qPCR. Data are presented as the mean ± S.D, ^*^p < 0.05, ^**^p < 0.01, n = 3. (**H**) The 5-Ethynyluridine (EU) labeling assay was used to determine APP mRNA stability under PARP16 knockdown. Cells were first treated with EU (0.1 mM) for 24 h, and the incorporation of EU was determined. Then, EU was removed, and cells were cultured in fresh media for another 0, 2, 4, 6, or 8 h. The stability of APP mRNA was determined as the percentage of labeled mRNA after the removal of EU divided by the percentage of labeled mRNA before removal. Data are presented as the mean ± S.D, ^*^p < 0.05, ^***^p < 0.001, n = 3. (**I**) RIP assay in HEK-APP cells demonstrating significant enrichment of APP mRNA using PARP16 antibodies compared to the negative control IgG. Data are presented as the percentage of input (± S.D, n = 3), ^***^p < 0.001. (**J**) The RNA pull-down assay in Aβ_1-42_-treated HT22 cells lysate using either the APP or mutated-APP RNA. GFP RNA was used as a negative control.

**Figure 6.**
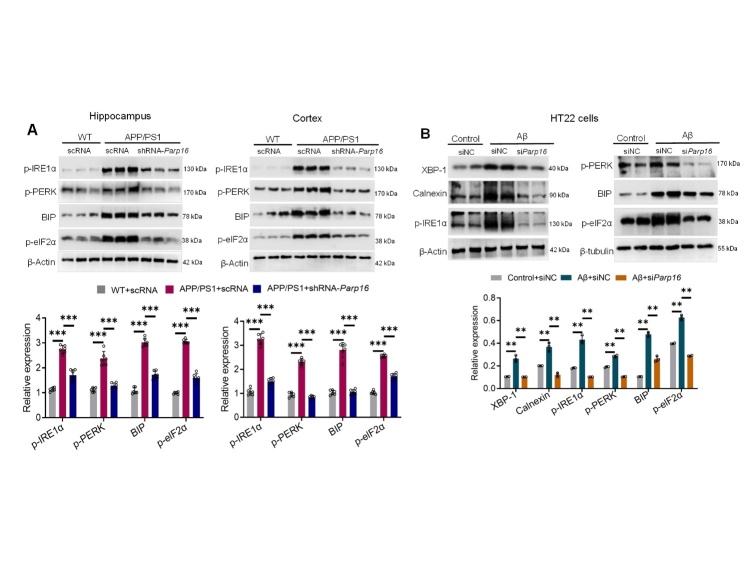
**PARP16 knockdown suppresses ER stress in APP/PS1 mice and Aβ-treated neuron cells**. (**A**) Knockdown of PARP16 attenuated ER stress by mediating the PERK and IRE1α signaling pathways in APP/PS1 mice. APP/PS1 and WT mice were injected intracerebroventricularly (i.c.v.) with an AAV-expressing shRNA targeting PARP16 (shRNA-*Parp16*) or AAV-scramble control shRNA (scRNA, used as a control). p-PERK and p-IRE1α signaling was determined by western blots in the hippocampi and cortices. Data represent the mean ± S.D. ^***^p < 0.001, n = 6. (**B**) PARP16 knockdown attenuated ER stress in Aβ_1-42_-treated HT22 cells. HT22 cells transfected with negative control (siNC) mimics or si*Parp16* were treated with or without Aβ_1-42_, and p-PERK and p-IRE1α signaling were measured by western blotting. Data represent the mean ± S.D, ^**^p < 0.01. At least three experiments were repeated.

### Knockdown of PARP16 decreases APP mRNA stability and expression

Next, we used the HEK/APPsw cell line, a well-established cellular model stably transfected with APP cDNA, to validate the PARP16 expression. There was a significant increase in PARP16 expression in HEK/APPsw cells compared to that in control HEK cells (Fig. 5A). Furthermore, we used HEK/APPsw cells transfected with control or *PARP16* siRNA to evaluate the effect of silencing PARP16 on APP expression. Successful transfection and efficient PARP16 silencing were confirmed using western blotting (Fig. 5B). Overexpression of APP leads to an elevation in APP levels, which is consistent with a previous report [34]. As expected, APP expression was decreased in siRNA *PARP16*-treated cells compared to that in control siRNA-transfected HEK-APP cells (Fig. 5B). We found that the protein level of APP decreased following the knockdown of PARP16 in HEK-APP cells. Therefore, we focused on APP expressions in order to understand how PARP16 regulates APP expression. First, we treated PARP16 knockdown-HEK-APP cells with proteasome or autophagy inhibitors, such as MG132, 3-MA, or NH_4_Cl. The level of APP did not recover after MG132, 3-MA, or NH_4_Cl treatment (Fig. 5C), implying that the reduced APP levels observed in PARP16 knockdown cells did not result from increased protein degradation. However, *APP* mRNA levels were significantly decreased in PARP16 knockdown HEK-APP cells (Fig. 5D). Therefore, we investigated whether PARP16 affects *APP* mRNA stability and transcription. We used a nuclear run-on assay to assess whether the reduced APP mRNA expression in PARP16 depleted cells was due to gene transcription. However, APP transcription was unchanged in PARP16 knockdown cells (Fig. 5E). Next, we assessed the effects of PARP16 on mRNA stability. To this end, we treated HEK-APP cells with the transcriptional inhibitor actinomycin D and assessed the mRNA levels of APP over time. The half-lives of mRNA were significantly shorter in PARP16 knockdown cells than in controls (Fig. 5F), while restoration of PARP16 expression restored APP mRNA levels (Fig. 5G). We further measured RNA stability using the 5-ethynyluridine (EU) labeling and release method. We consistently observed a significant decrease in EU-labeled *APP* RNA in PARP16 knockdown cells (Fig. 5H). Meanwhile, we used RIP to explore whether PARP16 may regulate mRNA stability by interacting with mRNA and observed that PARP16 could interact with *APP* mRNA (Fig. 5I). We performed an RNA pull-down assay using in vitro biotin labeling of *APP* mRNA and found that PARP16 had a strong binding affinity for *APP* mRNA in HT22 cells exposed to Aβ (Fig. 5J). Thus, these results suggested that PARP16 could interact with *APP* mRNA and stabilize *APP* mRNA, and knockdown of PARP16 could reduce APP expression, consistent with reduced amyloid aggregation in APP/PS1-shRNA-*Parp16* mice.

### Knockdown of PARP16 reduces ER stress

Increased ER stress has been shown in AD brains, so we investigated the effects of PARP16 deficiency on the ER stress sensors PERK and IRE1α in APP/PS1 mice and neurons. In agreement with the increased levels of PARP16, we also observed an increase in ER stress sensors p-PERK and p-IRE1α in both the hippocampi and cortices of APP/PS1 mice, but PARP16 knockdown reversed ER stress sensors (Fig. 6A). Consistently, we observed significant differences in the p-PERK and p-IRE1α pathways in HT22 cells treated with the Aβ peptide between the control and knockdown PARP16 cells (Fig. 6B). These results suggest the potential involvement of PARP16 in the regulation of the neuronal PERK and IRE1α pathways during the ER stress response. Next, we determined ER stress in HEK-APP cells. When the levels of the ER stress sensors PERK and IRE1α were analyzed by western blotting, we found that there were significantly increased levels in HEK-APP cells, but PARP16 knockdown reversed ER stress sensors (Supplementary Fig. 1A). In addition, we also observed that tauroursodeoxycholic acid (TUDAC), a pharmacological ER stress inhibitor, decreased ER stress sensors in a concentration-dependent manner (Supplementary Fig. 1B). These results suggest the potential involvement of PARP16 in the regulation of the neuronal PERK and IRE1α pathways during the ER stress response.

## DISCUSSION

The results of this study indicate that PARP16, a major transducer of ER stress signals, is upregulated in the brains of APP/PS1 transgenic AD mice, which enhances Aβ generation by regulating APP mRNA stability, and APP levels, resulting in neuropathogenesis. More importantly, knockdown of AAV-mediated PARP16 partly improved impaired spatial memory deficits and attenuated many AD-associated pathologies, including cell death, inflammatory responses, and ER stress, at least partly by decreasing Aβ accumulation mediated by the degradation of APP mRNA. Understanding the mechanism and function of PARP16 in AD pathology may provide important information for the development of novel therapeutic agents.

PARP16, a PARP family of enzymes, is a membrane-anchored ADP-ribosyltransferase that is associated with the endoplasmic reticulum [35]. Growing evidence suggests that the PARP family of enzymes regulates a wide range of molecular mechanisms and plays an important role in cellular functions [19]. Dysregulation of PARP activity triggered by diverse cellular stresses, including ER stress, leads to the regulation of mRNA stability and protein synthesis through post-transcriptional mechanisms, which can affect the disease state. For instance, PARP14 demonstrated important roles in DNA damage response, T-cell development, macrophage activation and tumor development [36, 37]. PARP10 was demonstrated to be a novel regulator of NF-κB signaling by directly modifying NEMO [38]. Likewise, our previous studies demonstrated that PARP16 plays an important role in vascular diseases through activation of PERK and IRE1α signaling in the UPR [21]. Here, we found that the level of PARP16 was positively linked to Aβ levels in three systems: in Aβ_1-42_-induced HT22 cells, in the brains of APP/PS1 mice, and in the HEK293 expressing APP cell line. To functionally test the relevance of chronic PARP16 activation in AD pathogenesis, we performed extensive studies using AAV-mediated knockdown of PARP16 in the APP/PS1 mouse model, in which abundant Aβ plaques and deficits in spatial learning were clearly visible. Knockdown of PARP16 impacted cardinal features of AD pathology, leading to (i) reduced amyloid deposits, (ii) improved spatial memory dysfunction, and (iii) attenuated neuroinflammation and cell apoptosis. Aβ and its oligomers are thought to be the major toxic components, Aβ stimulation induces cell injury, such as neuroinflammation and cell apoptosis. Similar to the in vivo studies, knockdown of PARP16 attenuated Aβ-induced neuronal injury by reducing apoptosis and inflammation. Taken together, our results suggest that the knockdown of PARP16 mediates some of the neuroprotective effects in Alzheimer's pathogenesis.

The amyloid cascade hypothesis is one of the most prominent theories in Alzheimer's disease research. APP is a transmembrane protein that plays a crucial role in Aβ peptide production. Familial AD mutations have been identified in the APP promoter region, which increases APP mRNA expression and is associated with an increased risk of AD [39], demonstrating that APP expression levels can have profound effects on AD pathogenesis. Additionally, APP mRNA and protein levels have been shown to be elevated in sporadic AD, correlating with elevated sAPPα and soluble Aβ [40]. In AD brains, specific APP mRNA splice variants are specifically elevated. These data show that increased APP provides more substrate for increasing Aβ generation and amyloid plaque deposition, which can accumulate over time to profoundly affect disease. mRNA is tightly regulated at the post-transcriptional level. Studies have shown that many PAPRs play key roles in RNA processing through various mechanisms, such as mRNA binding [19]. These results highlight the importance of the PARP family members in mRNA processing and protein abundance. Our results indicated that knockdown of PARP16 decreased APP mRNA levels. Further research demonstrated the binding affinities of PARP16 to APP RNA, causing it to be stable and translated, affecting APP production and processing into Aβ. In contrast, PARP16 knockdown decreased APP mRNA stability and inhibited APP levels. These observations are in agreement with previous findings that dysregulation of PARP activity leads to regulation of mRNA stability and protein expression. Collectively, our results uncover a novel output of PARP16 where it increases APP steady-state levels, in turn promoting APP mRNA stability and increasing APP protein abundance, possibly accelerating the amyloid cascade. Importantly, knockdown of PARP16 attenuated the increase in APP, reducing AD-associated pathologies.

On the other hand, ER stress has been increasingly implicated in the pathophysiology of AD [41, 42]. ER stress caused by the accumulation of unfolded and malfunctional proteins is a short-term adaptive response; however, if it persists and is not resolved, it leads to tissue dysfunction [43]. Prolonged ER stress results in cell death via apoptosis activation. PARP16 transduces ER stress through two components of the UPR, PERK and IRE1 signaling pathways. Similarly, we investigated ER stress responses and found that the pathological consequences of chronic PARP16 were linked to sustained ER stress. In our study, markers of ER stress were upregulated in the cortex and hippocampus of APP/PS1 mice, in line with the observations that ER stress is associated Alzheimer’s disease pathology and may potentiate cell loss in the disease [44]. APP expression is specifically associated with increased ER stress. Furthermore, preventing the upregulation of PARP16 results in decreased ER stress and cell injury. Collectively, it is likely that PARP16 promotes APP stabilization in neurons, acts synergistically to stimulate the UPR in neurons, and subsequently provokes AD pathology in the context of prolonged stress.

**Figure 7.**
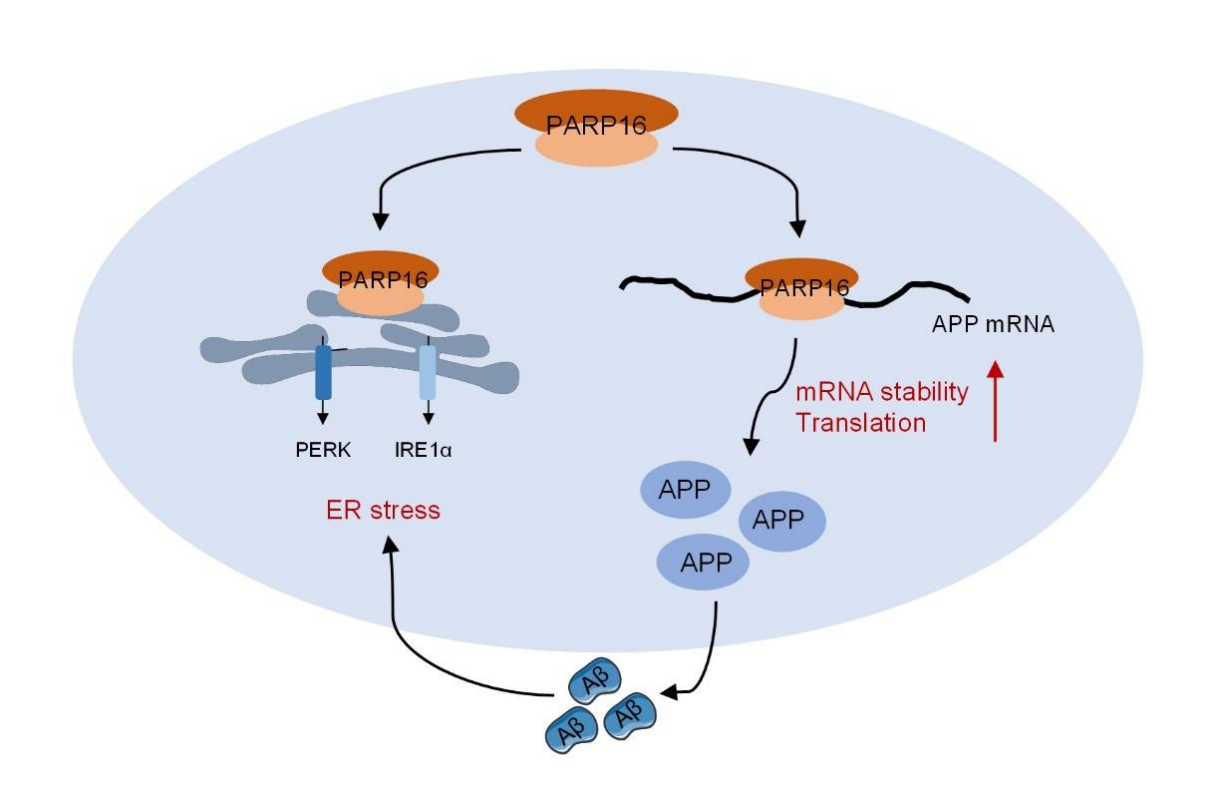
**Schematic representation of PARP16-mediated Alzheimer’s disease pathogenesis**. PARP16 is significantly increased in AD pathology and intracellular PARP16 acts as an RNA binding that regulates the mRNA stability of APP and protects targeted APP from degradation, thereby increasing APP levels and AD pathology.

## Conclusions

Taken together, as summarized in Figure 7, our study uncovered a previously unanticipated role for PARP16 in the stabilization of APP RNA. Our results suggest that the increased expression of PARP16 in AD pathology progression may be important not only in ER stress, but also in promoting APP stabilization. Therefore, targeting intracellular PARP16 to induce APP degradation may be a promising strategy to slow the progression of AD.

## Supplementary Materials

The Supplementary data can be found online at: www.aginganddisease.org/EN/10.14336/AD.2023.0119.
