# Supplementary material for: PARP16-Mediated Stabilization of Amyloid Precursor Protein mRNA Exacerbates Alzheimer’s Disease Pathogenesis
Source: Aging Dis. 2023 Aug 1;14(4):1458–71. doi: 10.14336/AD.2023.0119 (PMC10389827; doi:10.14336/AD.2023.0119)
Supplement: Supplementary file 1 — The Supplementary data can be found online at: www.aginganddisease.org/EN/10.14336/AD.2023.0119. [file AD-14-4-1458-s.pdf]

SUPPLEMENTARY DATA

PARP16-Mediated Stabilization of Amyloid Precursor Protein mRNA Exacerbates Alzheimer's Disease Pathogenesis

Jinghuan Wang, Qianwen Cheng, Yuyu Zhang, Chen Hong, Jiayao Liu, Xinhua Liu, Jun Chang

© 2023. Wang J et al. Published online at https://www.aginganddisease.org/EN/10.14336/AD.2023.0119

SUPPLEMENTARY DATA

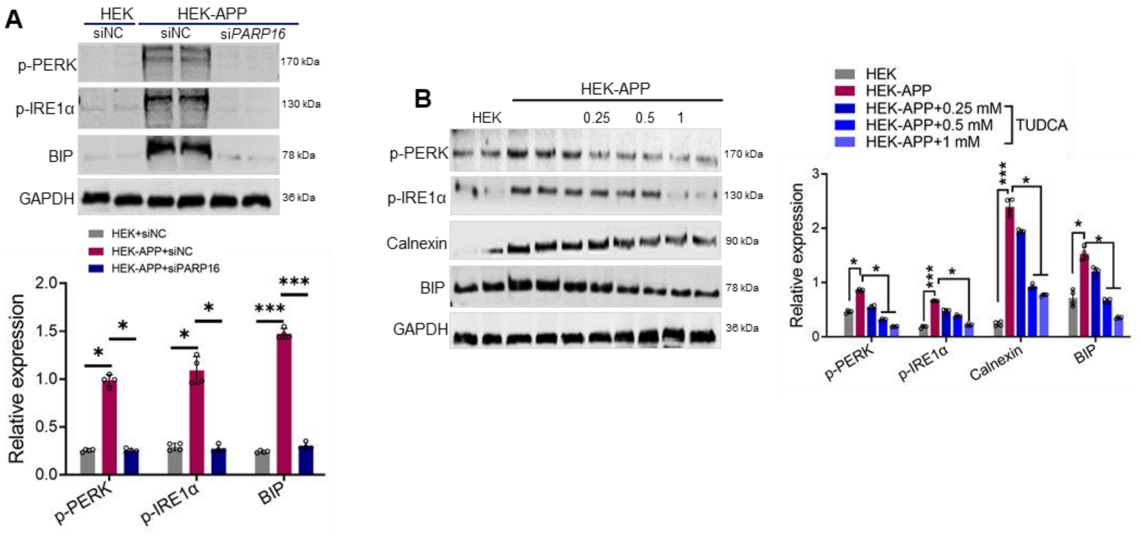

Supplementary Figure 1. TUDCA attenuated ER stress in HEK-APP Cells. (A) Knockdown PARP16 attenuated ER stress though mediating PERK and IRE1α signal pathway in HEK-APP Cells. HEK and HEK-APP cells were transfected with negative control (siNC) mimics or si*Parp16*, and p-PERK and p-IRE1α signaling were measured by Western blotting. **(B)** TUDCA attenuated ER stress in HEK/APPsw Cells. HEK and HEK-APP cells were treated with indicated concentrations TUDCA, and p-PERK and p-IRE1α signaling were measured by Western blotting. Data represent the mean ± SD, $^{*}p < 0.05$, $^{***}p < 0.001$, at least three experiments were repeated.

© 2023. Wang J et al. Published online at https://www.aginganddisease.org/EN/10.14336/AD.2023.0119
